# Online Quality Control of Powder Bed Fusion with High-Resolution Eddy Current Testing Inductive Sensor Arrays

**DOI:** 10.3390/s24216827

**Published:** 2024-10-24

**Authors:** Pedro Faria, Rodolfo L. Batalha, André Barrancos, Luís S. Rosado

**Affiliations:** 1 Strata Tech Labs, Rua da Bela Vista à Graça 27, 819, 1170-054 Lisbon, Portugal; pedrogsfaria@tecnico.ulisboa.pt; 2 Instituto de Soldadura e Qualidade, Avenida Professor Dr. Cavaco Silva 33, Taguspark, 2740-120 Porto Salvo, Portugal; rlbatalha@isq.pt; 3 Instituto Superior Técnico, University of Lisbon, Av. Rovisco Pais 1, 1049-001 Lisbon, Portugal; andre.barrancos@tecnico.ulisboa.pt; 4 Instituto de Telecomunicações, Av. Rovisco Pais 1, 1049-001 Lisbon, Portugal

**Keywords:** additive manufacturing, powder bed fusion, eddy current testing, inductive sensor arrays

## Abstract

This paper presents the development of a novel eddy current array (ECA) system for real-time, layer-by-layer quality control in powder bed fusion (PBF) additive manufacturing. The system is integrated into the recoater of a PBF machine to provide spatially resolved electrical conductivity imaging of the manufactured part. The system features an array of 40 inductive sensors spaced at 1 mm pitch and is capable of performing a full array readout every 0.192 mm at 100 mm/s recoater speed. Array scalability was achieved through the careful selection of the electromagnetic configuration, miniaturized and seamlessly integrated sensor elements, and the use of advanced mixed signal processing techniques. Experimental validation was performed on stainless steel 316L parts, successfully detecting metallic structures and confirming system performance in both laboratory and real-time PBF environments. The prototype achieved a signal-to-noise ratio (SNR) of 26.5 dB, discriminating metal from air and thus demonstrating its potential for improving PBF part design, process optimization, and defect detection.

## 1. Introduction

Additive manufacturing (AM) has undergone considerable changes since its original application in prototypes in the 1980s [1]. The process’s advantages are that it is incredible for the fabrication of complex geometries with high precision, providing reduced material waste, design flexibility, and customization [2]. Metal AM processes have become increasingly popular across industries like aerospace (e.g., turbine blades [3]) and biomedicine (e.g., metal implants [4]), where complexity and weight requirements are too demanding for traditional manufacturing methods.

Powder bed fusion (PBF) is one of the most prominent AM techniques for 3D printing metallic parts [1]. The energy of a laser beam or an electron beam melts specific regions of successively processed powder layers [3]. Once a layer is melted, a recoater blade spreads the powder for the next layer. Despite impressive evolution so far, enhanced process robustness, repeatability, and stability is needed to produce critical parts. In fact, process deviation can occur, resulting in unexpected defects such as pores, high surface roughness, cracking, and delamination, among other occurrences [5]. Conventional non-destructive testing (NDT) is applied as a post-production quality control (QC) step, with remarkable limitations concerning the type, severity, and location of the defective conditions that can be detected [6,7].

On the other hand, in situ layer-wise imaging may provide QC information across the entire part volume, including on features and surfaces that become later inaccessible, in real time with the production, as an alternative to post-production NDT procedures [8]. So far, different methods have been used for in situ QC like optical sensors used to assess the melt pool stability [9], Spatially Resolved Acoustic Spectroscopy (SRAS) used to identify surface and subsurface defects [10], and laser-assisted ultrasounds to generate and detect waves resulting in 2D images [11].

The previously reported methods rely on the analysis of process signatures, therefore providing an indirect assessment of defective conditions. Alternatively, the assessment of effectively consolidated metal can provide direct visibility over defective conditions. Eddy current testing (ECT) is a popular NDT method used to inspect electrically conductive materials [12]. ECT relies on magnetically inducing and sensing electrical currents in the part under inspection. The interaction between the magnetic field and the part characteristics (e.g., defects, porosity) leads to changes in the material magnetic field which can be sensed by inductive or other sensor types.

**Figure 1:**
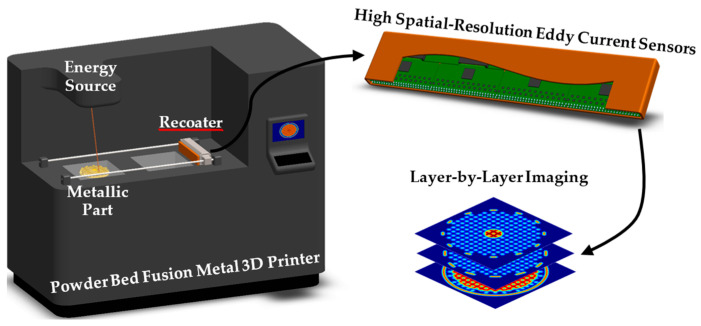
Powder bed fusion quality control with eddy current sensors.

Research progress has been made, with single ECT sensors already having been demonstrated together with commercial PBF machines [13,14,15]. Nonetheless, a sensor array is key for surpassing most of the found limitations while capturing more information in a single pass, drastically improving detection speed, efficiency, accuracy, and reliability [16]. ECT sensor arrays can be integrated with PBF, as illustrated in Figure 1, to provide spatially resolved electrical conductivity imaging with a spatial resolution constrained by the sensor’s size and pitch and the array readout speed [7]. Relying on the difference in electrical conductivity between raw powder and consolidated metal, a layer-by-layer reconstruction of the produced part can be computed afterwards by applying machine learning algorithms [17].

Research into ECT array probes aims to increase the spatial resolution, which implies the exploration of different sensing elements and acquisition methods. Some developments allow for the optimization of the resolution and speed by using a multi-channel approach [18,19] or multiplexing [20,21]. The challenge of these implementations is the inherent trade-off between resolution and readout speed. In [22], an array of 128 magneto-resistive (MR) sensors was used to achieve a 135 μm scan resolution while constraining the recoater speed to 24 mm/s.

This paper continues the effort made in [23], solving previous limitations and improving the overall system specifications. The newly developed eddy current array (ECA) system operates by being mounted on the PBF printer recoater, allowing layer-wise imaging to entirely reconstruct the metal part under production. The enhanced design allows for a spatial resolution of 1 mm, a sampling frequency of 31.25 kHz, and an excitation frequency of 1 MHz and achieves scan resolutions as low as 0.192 mm at the nominal 100 mm/s recoater speed.

## 2. System Hardware

### 2.1. Top-Level Architecture

**Figure 2:**
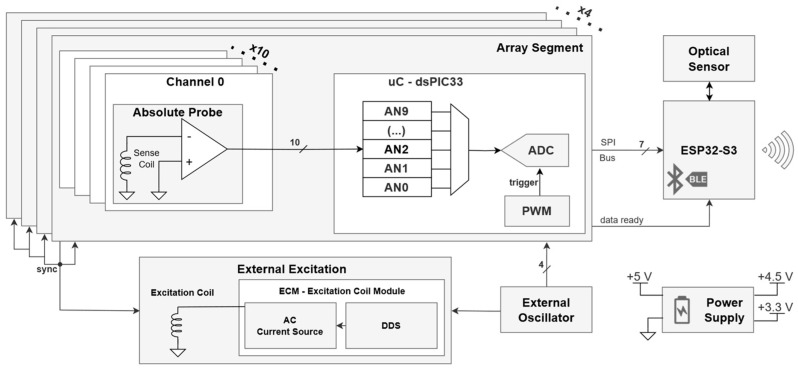
Eddy current array monitoring system block diagram.

The developed ECA system makes several improvements to allow the implementation of EC sensor arrays widely enough to assess the complete build surface of PBF machines with sub-mm sensor pitch capability. The overall architecture of the proposed system is presented in Figure 2.

Unlike traditional ECA instruments, the proposed system explores minimalist analog front-end circuits with the goal of minimizing the implementation footprint, power dissipation, and temperature-related drifts alongside. This is possible given the chosen EC excitation and sensing electromagnetic elements discussed ahead. The produced proof-of-concept system includes a total of 40 sensing elements, spaced with 1 mm pitch.

Signals from each group of 10 sensing elements are digitally acquired and processed by one of the four Microchip dsPIC33 microcontrollers. Digitalized EC signals are read through SPI by an ESP32-S3 module board and finally transmitted to a personal computer through Bluetooth Low Energy (BLE).

### 2.2. Excitation and Sensor Elements

The proposed EC sensor array relies on a reflection topology, where a single wide coil creates a strong and uniform alternating magnetic field, and miniaturized sensitive coils assess the magnetic field changes. By using this arrangement, the magnetic field at each sensing coil is mostly the same, and by decoupling the excitation from the receiving coils, it allows the excitation coil to be optimized in terms of power consumption and its magnetic field to be increased, allowing the possibility to reduce the size of the sensing coils. This design decision also allows the use of a single current source circuit which is optimized for the generation of high excitation amplitude. Besides allowing for the individual optimization of those two elements, this also creates several optimization opportunities on the amplifier circuit necessary to handle the sensitive coil signals.

The excitation element is essentially a three-wire coil processed in the same printed circuit board (PCB) where the sensing coils are assembled. When driven with an alternating current, this element creates a primary magnetic field responsible for the induction of EC on the consolidated metal present underneath. The induced electrical currents predominantly follow the orientation of the excitation element and suffer modifications in agreement to the shape and the presence of imperfections on the surface of the part being tested.

**Figure 3:**
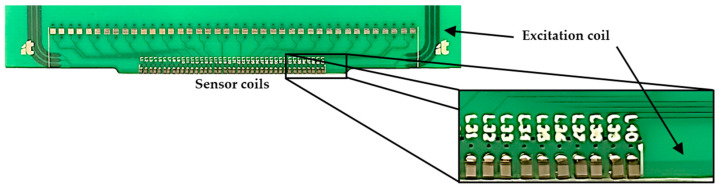
Sensing coils and excitation element coil.

The array of 40 sensing coils is responsible for assessing EC changes while probing the resulting magnetic field. Commercially available discrete surface-mounted device (SMD) coils, with dimensions of 1 mm × 0.5 mm × 0.5 mm and 10 μH inductance with a ferrite core, were used, providing low-cost and seamless integration with the excitation element and readout circuitry. Figure 3 shows the PCB integrating the excitation element and the sensing coils.

Frequency has an important effect on EC as it determines how the induced eddy current concentrations evolve from the surface down. For the present application, and to successfully assess the consolidation of successive layers, a substantial EC concentration is desired in the preceding layers. With stainless steel 316L (1.32 × 10^6^ S/m, 2.25 IACS, εr ≈ 1), a 1 MHz operation frequency leads to a standard depth of penetration (depth at which EC is reduced to 1⁄e) of around 400 µm, which accommodates multiple layers whose thickness usually ranges between 30 µm and 100 µm. In another aspect, this substantially high operation frequency allows a proper signal-to-noise ratio to be reached, even with the tiny SMD coils used.

### 2.3. Excitation Driver Circuit

Traditionally, a constant-amplitude sinusoidal voltage source is used to drive the excitation element, allowing the current amplitude to vary with the driven impedance. As the excitation element impedance varies with the consolidated metal presence or absence along a scan, the primary magnetic field would unwantedly vary accordingly. To solve that, the proposed system explores a voltage-controlled current source with high output capability, keeping the current amplitude constant regardless of the driven impedance changes.

**Figure 4:**
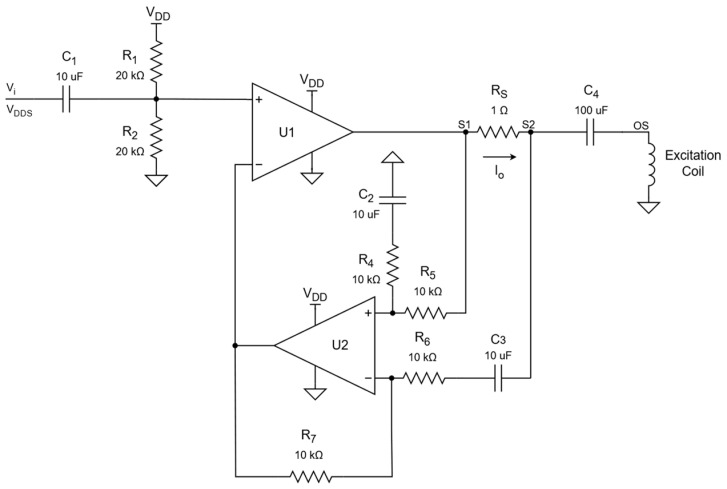
Excitation driver circuit schematic.

An Analog Devices AD9838 Direct Digital Synthesis (DDS) device was used to generate a sinusoidal signal with 10-bit amplitude resolution with 1 MHz frequency. The sinusoidal output voltage of the DDS is fed to the voltage-controlled current source. This current source is composed of a high-power Operational Amplifier (OPAMP) driver, a shunt resistor, and a subtractor circuit to provide current feedback, as shown in Figure 4.

Resistors $R_{1}$, $R_{2}$ and the subtractor circuit (U2, $R_{4}$, $R_{5}$, $C_{2}$, $R_{6}$, $R_{7}$, $C_{3}$) DC unitary gain ensure the polarization of the main driver (U1) at mid-supply. The AC gain of the subtractor is also unitary, which leads to a transconductance gain as follows:

(1) $$g_{m}=\frac{I_{O}}{V_{DDS}}=\frac{1}{R_{S}}=1\;A/V.$$

Impedance changes will therefore be corrected adjusting the driver output voltage within the operational limits. The output current limit of the circuit is around 500 mA; to limit the verified distortion, the DDS is set to generate a 350 mV amplitude output voltage.

### 2.4. Sensor Amplifier Circuit

**Figure 5:**
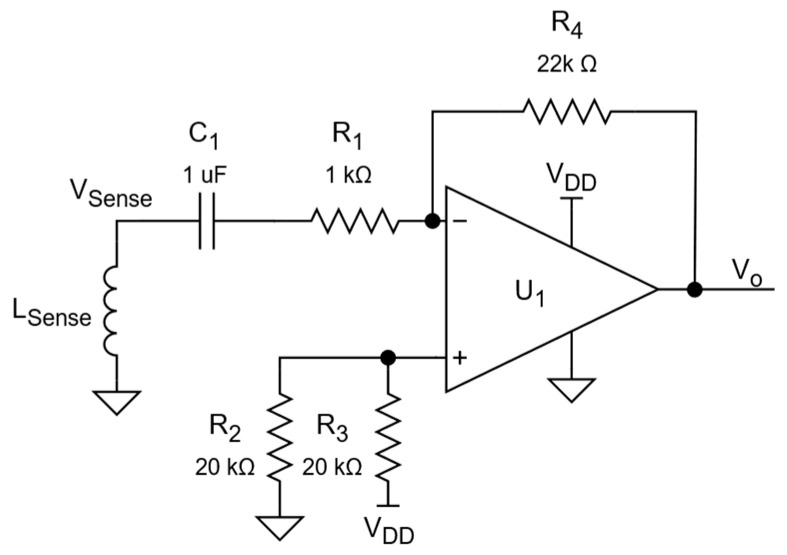
Sensor amplifier circuit schematic.

Even with a high-amplitude current flowing in the excitation element, the output voltage of the sensitive coils is substantially weak. To ensure the proper digital conversion, a 22× gain amplifier is used with each coil, as shown in Figure 5. Single-supply operation is achieved using AC coupling and ensuring bias at mid-supply.

### 2.5. Prototype and Specifications

**Figure 6:**
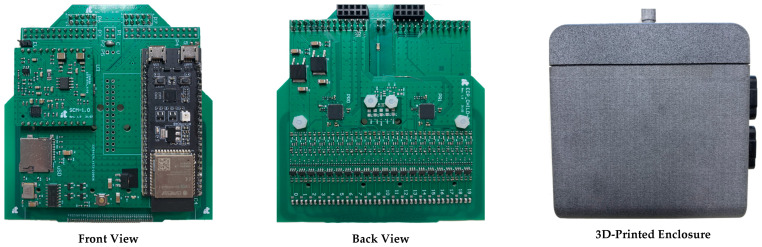
Eddy current array monitoring system PCB and 3D-printed enclosure.

The ECA system was prototyped with the development of PCBs for the several sub-systems, as shown in Figure 6. A 3D-printed enclosure to attach the ECA system to the recoater was also developed. The overall system specifications are listed in Table 1.

## 3. System Software

### 3.1. Digital Signal Processing

In addition to the signal conditioning realized by the sensor amplifier circuit, several signal processing tasks are performed at the digital level in the dsPIC33 microcontrollers. Throughput is enhanced by handling most of the operations on the microcontrollers’ hardware peripherals.

Demodulation and digital acquisition are performed by synchronously digitalizing the amplified signal at an arbitrary and constant instantaneous phase. A Moving Average Filter (MAF) is then used together with decimation to increase bit resolution and lower the sampling rate. The process is similar to the single-phase coherent demodulation previously used in [23]. Similarly, to achieve maximum sensitivity, the sampled instantaneous phase can be adjusted with 1° resolution.

**Figure 7:**
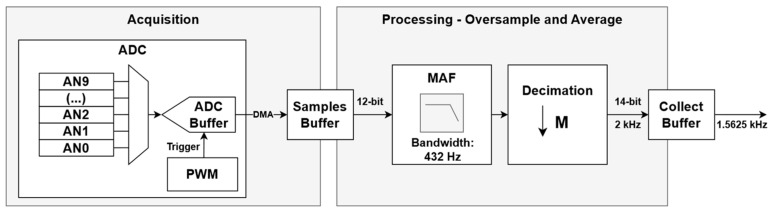
Digital signal processing performed using the dsPIC33 microcontrollers.

As depicted in Figure 7, the described processing is articulated in three main steps: the ADC samples and stores the ten analog channels; the available Direct Memory Access (DMA) triggers and transfers the data to a sample buffer; the CPU performs MAF operations and stores the result in a collect buffer.

### 3.2. Data Interface

**Figure 8:**
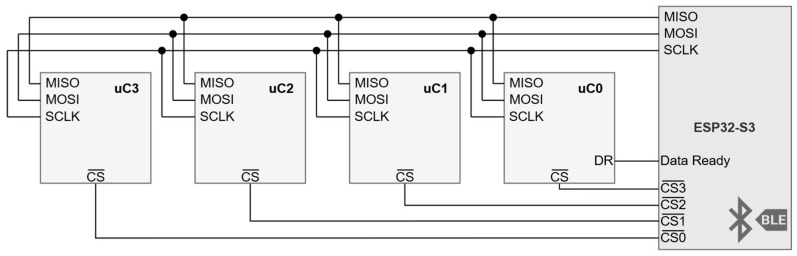
Data interface between the ESP32-S3 and the several dsPIC33 microcontroller instances.

The ESP32-S3 receives the processed samples from the several dsPIC33 microcontrollers though a Serial Peripheral Interface (SPI). As shown in Figure 8, a single bus clocked at 16 MHz is shared across the several instances, activated by an individual Chip-Select signal.

### 3.3. Wireless Interface

The Wireless interface is implemented, profiting from the capabilities of ESP32-S3. The architecture uses the FreeRTOS operating system kernel to manage the necessary concurrent threads. The ESP32-S3 is set to devices’ roles using the Generic Access Profile (GAP) capabilities. The Generic Attribute (GATT) services table is configured to activate Receiver (RX) and Transmitter (TX) capabilities. On top of this, the solution incorporates a third-party Python library called ble-serial 2.8.0 on the computer side. This library facilitates the selection of specific characteristics and maps them onto the appropriate COM Port channel.

A command-based protocol was established to orchestrate the system operation. Additionally, given BLE’s inherent low data throughput, firmware was developed to use a micro-SD card as a temporary buffer for acquired samples.

### 3.4. User Interface

**Figure 9:**
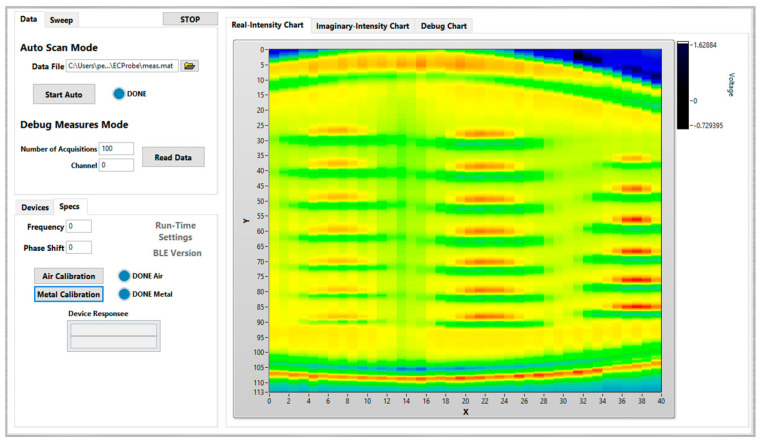
Eddy current array monitoring system user interface.

A sophisticated computer application was developed in LabVIEW to serve as the user interface, as shown in Figure 9. Besides configuring and controlling the system, this application includes the capability to control mechanized scan devices, used to initially validate and tune the probe under controlled laboratorial conditions.

## 4. Experimental Demonstration

The ECA system was initially validated under laboratorial conditions before being deployed into a real PBF machine.

### 4.1. Laboratorial Validation

The probe was mounted in a mechanized scan device capable of scan speeds up to 160 mm/s. A laptop ran the developed LabVIEW user interface application, used for data visualization and recording.

**Figure 10:**
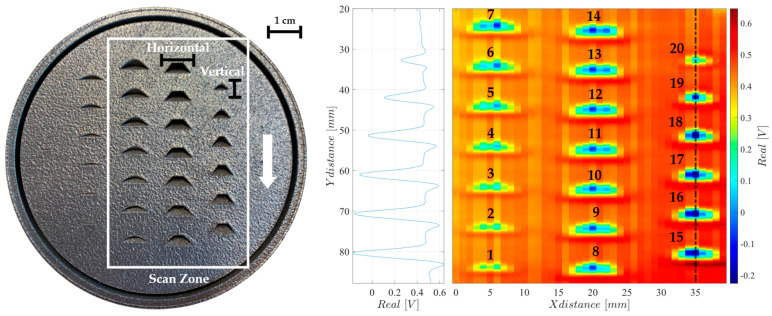
Two-dimensional imaging result on a two-dimensional scan over part 1.

**Figure 11:**
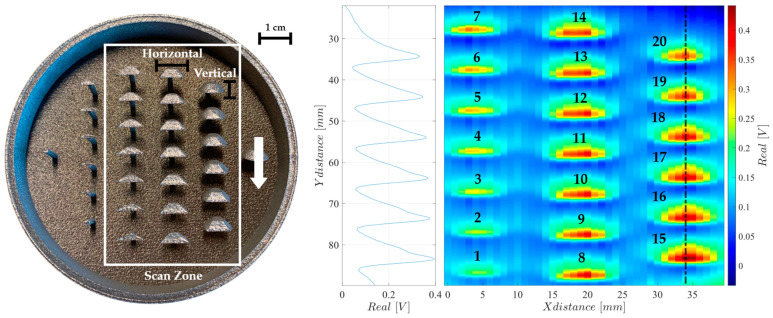
Two-dimensional imaging result on a two-dimensional scan over part 2.

Two PBF parts were produced in stainless steel 316L with a RenAM 500S Flex Laser Beam PBF machine at Instituto de Soldadura e Qualidade (ISQ) to emulate diverse shapes and sizes of a similar structure in order to understand the ECA’s reconstruction capability for the different features. These parts include a frustum cone positioned at different heights emulating its production at different layer stages. Part 1, illustrated in Figure 10, has high metal density and includes negative structures, while part 2, in opposition, features positive metal structures, as depicted in Figure 11. Both Figure 10 and Figure 11 show the signal’s real component, since the signal’s complex part is close to zero.

Imaging results for part 1 are also shown in Figure 10, where the contours of the metallic surfaces can be easily identified. Additionally, it is possible to verify an apparent increase in amplitude with the increase in the *y*-axis, which relates to the high depth of the structures. Some mismatches can be identified across the several channels, yet, with the verified high sensitivity, its effects are minimal.

Results for part 2, in Figure 11, show higher intensity in the superficial metal and a fadeout effect representing the depth of the structures. However, there is a decrease in sensitivity over the metal structures compared to the previous negative structures. This results from a weaker EC induced in such small-size structures.

Table 2 shows the size of the features in parts 1 and 2 and their reconstructed dimensions. From Table 2, it is clear how the system’s resolution is much higher in the vertical measurements when compared to the horizontal ones. This happens because of ECA’s high-frequency sampling rate along the Y axis, while the X axis resolution is linked to the 1 mm spacing between the coils.

For the vertical measurements, the ECA probe reconstructions have deviations of 0.14 mm for part 1 and 0.57 mm for part 2, when compared with the real vertical dimensions of each structure. Meanwhile, for the horizontal measurements, the deviations are 1.45 mm for part 1 and 2.41 mm for part 2. To calculate the horizontal and vertical sizes of each structure, the distance between the closest points from the reconstructed data was measured, where the sensor response dropped 60% from its maximum value.

### 4.2. Deployment in a PBF Machine

**Figure 12:**
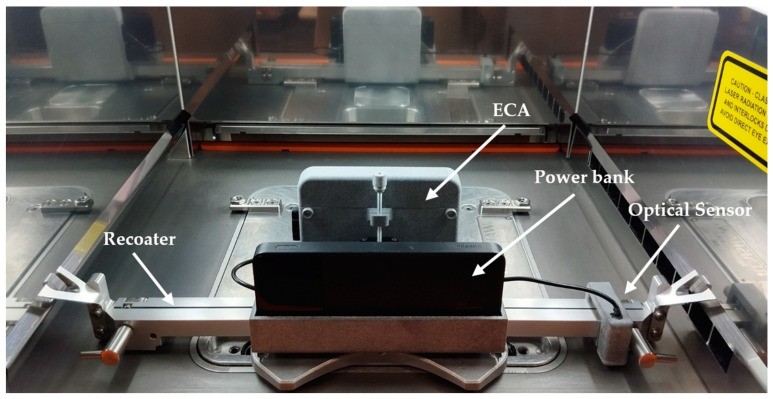
ECA system deployed in a RenAM 500S Flex PBF machine.

The ECA monitoring system was finally deployed in a RenAM 500S Flex PBF machine, as shown in Figure 12. The machine was configured with the reduced build volume accessory which reduced the processing area to 78 × 78 × 50 mm^3^.

A USB power bank was used to supply the ECA system, allowing operation for over 20 h. To ensure that data from successive layers were drawn with 3D coherency, an optical sensor was used and marking stripes were installed in the machine chassis. Also, the ECA system was set up with a lift-off distance of 0.1 mm.

**Figure 13:**
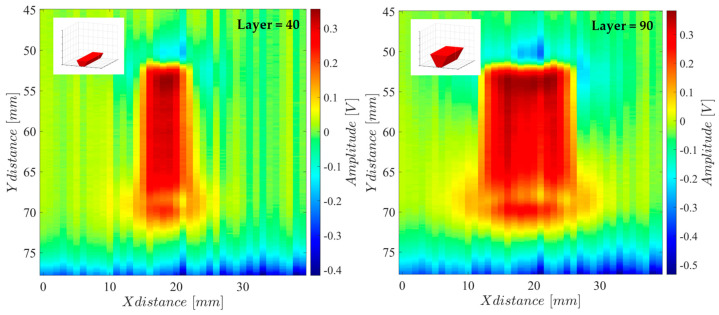

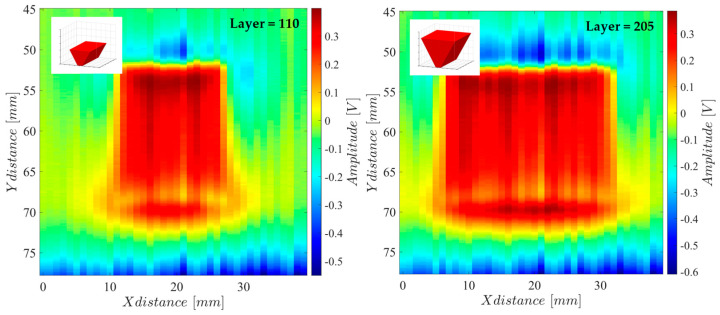
Real-time imaging results of several layers of the PBF-produced part.

A final validation test was performed with the ECA system performing measurements on the successive layers of a produced part in real-time. This part, with a trapezoidal shape produced with the smaller face facing down, is usually used for tunning PBF operational parameters. The imaging results for some of the processed layers are depicted in Figure 13. An SNR of around 26.5 dB was determined from the Figure 13 results, comparing the metal presence signal indication with the background noise.

## 5. Conclusions

This paper presented a new ECA system used to provide layer-by-layer and real-time imaging during the PBF process. Array scalability and high spatial resolution was achieved with the strategical selection of the electromagnetic configuration, a seamless integration between the sensor elements and the readout electronics, and advanced mixed-signal signal processing. The achieved results were only possible by an holistic approach which resulted in specifications that could not be met by the individual optimization of the sensor and the readout electronics.

The developed prototype system was successfully deployed in a PBF machine operation and demonstrated no limitations to its regular operation. Imaging results were gathered at a 100 mm/s recoater speed with a 1 mm spatial resolution in the recoater direction and 0.192 mm along the recoater sweep direction. Overall, the system has shown a high SNR of 26.5 dB. This promising result is an important step towards new methodologies for PBF part design and process optimization, quality control, and fault recovery.

Future work will focus on further improving the electromagnetic geometry to provide further sensitivity and effective resolution and to use coils with smaller pitches and sizes. The excitation current source will also be improved with an extended amplitude capability to compensate for the expected lower sensitivity of smaller sensor coils. Convolutional neural networks are under consideration firstly for the reconstruction of effectively produced parts and secondly for the direct detection of defective conditions. Also, interleaving is being considered to increase the spatial resolution along the recoater direction. The use of even-lower-dimension SMD coils is being studied for the same purpose with the difficulty of the much lower expected output voltages and attainable SNRs. A 240 mm wide probe is already being developed, targeting operation with a full-sized PBF machine build volume.

## Figures and Tables

**Table 1:** ECA system overall specifications.

Dimensions	100 mm × 100 mm
Sensor array width (coil span)	40 mm
Spatial Resolution (coil pitch)	1 mm
Scan resolution (at 100 mm/s recoater speed)	0.192 mm
Output sampling rate	31.25 kHz
Output resolution	14 bits
Excitation frequency	1 MHz
Supply voltage	5 V
Power consumption	2.748 W

**Table 2:** Part 1 and 2’s real and scanned structure sizes.

Features	Horizontal Size [mm]	Scan Part 1 Horizontal Size [mm]	Scan Part 2 Horizontal Size [mm]	Vertical Size [mm]	Scan Part 1 Vertical Size [mm]	Scan Part 2 Vertical Size [mm]
1	8.96	7.00	4.00	2.04	2.08	3.52
2	9.27	7.00	5.00	2.29	2.40	3.04
3	9.52	8.00	5.00	2.54	2.72	2.88
4	9.72	8.00	6.00	2.80	2.88	2.24
5	9.86	8.00	6.00	3.05	3.04	2.24
6	9.95	8.00	6.00	3.31	3.36	2.24
7	10.00	8.00	6.00	3.53	3.36	2.24
8	10.00	9.00	7.00	3.46	3.20	2.40
9	9.94	8.00	7.00	3.46	3.36	2.72
10	9.84	8.00	7.00	3.46	3.36	2.88
11	9.68	8.00	7.00	3.46	3.52	3.20
12	9.48	8.00	8.00	3.46	3.52	3.52
13	9.22	8.00	8.00	3.46	3.52	3.52
14	8.89	8.00	8.00	3.46	3.52	3.48
15	8.50	7.00	7.00	3.46	3.52	3.68
16	8.03	7.00	7.00	3.46	3.36	4.00
17	7.47	7.00	7.00	3.46	3.36	3.20
18	6.79	6.00	6.00	3.46	3.20	3.04
19	5.95	5.00	5.00	2.68	2.88	2.56
20	4.88	4.00	4.00	1.73	2.56	2.24

## Data Availability

The data presented in this study are not publicly available at this time but may be obtained from the authors upon reasonable request.
